# miR-139-5p Loss-Mediated WTAP Activation Contributes to Hepatocellular Carcinoma Progression by Promoting the Epithelial to Mesenchymal Transition

**DOI:** 10.3389/fonc.2021.611544

**Published:** 2021-04-15

**Authors:** Wenli Liu, Xuewei Gao, Xiaolong Chen, Na Zhao, Ying Sun, Yawen Zou, Yize Guan, Lin Yang, Xiaoxian Pei, Guozhen Wang, Bin Wang, Mingcheng Li, Wengang Song

**Affiliations:** ^1^Clinical Laboratory Diagnostics, Medical Technology College, Beihua University, Jilin, China; ^2^Department of Infectious Diseases, The First Affiliated Hospital of Zhengzhou University, Zhengzhou, China; ^3^Institute of Military Veterinary Medicine, Academy of Military Medical Sciences, Changchun, China; ^4^School of Medicine, Beihua University, Jilin, China

**Keywords:** WTAP, metastasis, HCC, epithelial to mesenchymal transition, miR-139-5p

## Abstract

**Background:** Hepatocellular carcinoma (HCC) is a primary aggressive gastrointestinal neoplasm that affects patients worldwide. It has been shown that Wilms' tumor 1-associating protein (WTAP) is frequently upregulated in various cancers. However, the potential role of WTAP in HCC remains largely unknown.

**Methods:** The expression levels of WTAP in human HCC tissues were determined by the western blotting and immunohistochemical (IHC) staining. A correlation between the WTAP expression, clinicopathological features, and the HCC prognosis was analyzed. The WTAP expression was silenced by short hairpin RNA (shRNA), and effects of the knockdown of WTAP on the proliferation and invasion of HCC cells were assessed. The microRNAs (miRNAs) involved in the regulation of the WTAP expression were identified by a bioinformatics analysis and further confirmed by *in vitro* assays.

**Results:** The expression levels of WTAP in liver cancer tissues were significantly elevated and compared with those in the adjacent normal tissues and significantly correlated with the clinical stage and prognosis in patients with HCC. Further investigation revealed that the knockdown of WTAP drastically suppressed HCC cell proliferation and invasion abilities. Luciferase reporter assay and validation experiments confirmed that WTAP was a direct target of miR-139-5p. Moreover, the overexpression of WTAP could partly abolish the inhibitory effects of miR-139-5p on the HCC cell growth and invasion. Mechanistically, we revealed that the miR-139-5p/WTAP axis regulated the HCC progression by controlling the epithelial to mesenchymal transition (EMT).

**Conclusions:** In summary, the results indicate that WTAP is a potential oncogene in HCC and miR-139-5p negatively regulates the WTAP expression. MiR-139-5p/WTAP can be utilized as a potential therapeutic target for HCC.

## Background

Hepatocellular carcinoma (HCC) is a primary aggressive and malignant gastrointestinal tumor that affects patients worldwide and is associated with high rates of morbidity and metastasis (1). Although a great progress has been made in diagnostic and therapeutic methods, the clinical outcome of most patients with HCC remains poor (2). A poor outcome of HCC therapy is largely due to a high incidence of its recurrence and metastasis (3, 4). Previous studies have shown that the epithelial to mesenchymal transition (EMT) process, during which epithelial cells acquire new features of mesenchyme, is a crucial mechanism that contributes to the recurrence and metastasis of cancer. The EMT process is characterized by an upregulation of the MMP family (MMP2/7/9) and epithelial markers (N-cadherin, vimentin) as well as a decrease of mesenchymal markers (such as E-cadherin) (5). However, a precise molecular mechanism underlying the HCC metastasis is little known. Thus, an improvement in understanding the key molecules in EMT processes is urgently needed.

The Wilms' tumor 1-associating protein (WTAP) gene located in the chromosomal region 6q25-27 was first identified in 2000 (6). WTAP is a nuclear protein that is ubiquitously expressed in a variety of human tissues. Recent studies have shown that WTAP functions in various physiological and pathological processes by regulating the cell cycle, proliferation, apoptosis, RNA splicing, and N6-methyladenosine RNA modification (m6A) (7–13). In addition, an increasing evidence has confirmed that WTAP plays crucial ontogenetic roles in various cancers, including gastric cancer, acute myelogenous leukemia, cholangiocarcinoma, colorectal cancer, glioma, pancreatic cancer, and renal cell carcinoma (14–20). Chen et al. (21) reported that WTAP promotes the progression of HCC in a m6A-dependent manner. Nevertheless, whether the expression of WTAP in HCC is associated with the progression of malignancy is still largely unknown.

Previous studies have demonstrated that microRNAs (miRNAs), a group of non-coding RNA molecules, are involved in many biological processes, such as cell proliferation, migration, inflammation, and apoptosis. Additionally, miRNAs play important roles in the process of tumorigenesis by acting as oncogenes or tumor suppressors (22, 23). Among these miRNAs, miR-139-5p, which is located in the chromosomal region 11q13, has been shown to be involved in many human tumors including HCC (24, 25). It has been reported that a decreased miR-139-5p expression in HCC is correlated with a poor prognosis (26). In addition, Wong et al. (26) found that miR-139-5p can inhibit the proliferation of HCC cells by regulating Rho-kinase 2. Moreover, miR-139-5p attenuates the HCC progression by targeting TCF-4 (27). These findings indicate a critical role for miR-139-5p in the development of HCC.

In this study, we investigated the potential molecular mechanisms of WTAP in the HCC progression. We found that an elevated WTAP in HCC may serve as an independent biomarker for prognosis. The knockdown of WTAP drastically impaired HCC cell proliferation and invasion *in vitro* and tumor growth *in vivo*. In addition, we revealed that WTAP promoted the activation of EMT in HCC. Moreover, miR-139-5p attenuated the WTAP expression through a direct binding of the 3′ untranslated region (3′-UTR) of WTAP messenger RNA (mRNA) and exercising its tumor-suppressive effects. Altogether, these findings identify a potential biomarker and target for the HCC diagnosis and therapy.

## Materials and Methods

### Clinical Samples and Cell Lines

A total of 341 human HCC samples and the corresponding normal liver tissue samples were obtained as previously described, after we obtained a written informed consent, according to an established protocol approved by the Ethics Committee of Zhengzhou University. HEK293T cells and HCC cell lines (SMMC-7721 and Hep3B cells) were purchased from Guangzhou Gino Bio Co., Ltd. (Henan, China). The cells were cultured in a Dulbecco's modified Eagle's medium (Gibco, MA, USA) supplemented with 10% fetal bovine serum (Gibco, MA, USA).

### Public Data Set Analysis

Public data sets were analyzed based on The Cancer Genome Atlas (TCGA) and Gene Expression Omnibus (GEO) databases as described in a previous study (28).

### Quantitative Real-Time PCR

The total RNA from HCC tissues or cell lines was extracted by using TRIzol (Invitrogen, CA, USA), as instructed by the manufacturer. Complementary DNA (cDNA) was synthesized by using the Superscript III Reverse Transcription Reagent (Life Technologies, CA, USA). Quantitative real-time PCR (qRT-PCR) was performed by using the TaqMan Power SYBR Green PCR Mix kit (Invitrogen, CA, USA) on an Applied Biosystems 7500 (Applied Biosystems, MA, USA). The expression level of miR-139-5p was normalized to the expression level of U6 by using the 2^−ΔΔ^CT method.

### Transfection of miRNAs and Small Interfering RNAs

SMMC-7721 or Hep3B cells (1 × 10^6^ cells/well) were seeded into a 6-well plate. About 24 h later, the cells were transiently transfected with a miR-139-5p mimic or inhibitor, short hairpin RNA (shRNA) targeting WTAP, or the corresponding negative controls (NCs) by using Lipofectamine 3000 (Invitrogen, CA, USA) based on the manufacturer's protocol.

### Cell Counting Kit-8 Assay

The cell proliferation was measured by using the cell counting kit-8 (CCK-8) assay at 1, 2, 3, and 4 days after the transfection. The cells were seeded in a 96-well plate at a density of 1,500 cells/well, and 10 μl of CCK-8 was added to 90 μl of the culture medium per well. The cells were incubated for 2 h, and the cell viability was measured as the absorbance at 450 nm by using a microplate reader (Bio-Tek Instruments Inc., VT, USA).

### Wound Healing Assay

For the evaluation of cell migration ability, a wound healing assay was performed as described in a previous study (29). The details were listed in the Supplementary Methods.

### 5-Ethynyl-2′-Deoxyuridine Incorporation Assay and Colony Formation Assay

Detailed information about the ethynyl-2′-deoxyuridine (EdU) assay and colony formation assay was provided in the Supplementary Methods.

### Western Blot Assay

For western blotting, the proteins in the cell lysates were separated by using an electrophoresis in an safety data sheet-(SDS-) polyacrylamide gel and electrophoretically transferred to a polyvinylidene difluoride (PVDF) membrane, which was sequentially incubated with primary antibodies against WTAP (60188-1-Ig, Proteintech, Hubei, China), E-cadherin (20874-1-AP, Proteintech, Hubei, China), N-cadherin (22018-1-AP, Proteintech, Hubei, China), Slug (ab106077, Abcam, MA, USA), Snail (ab53519, Abcam, MA, USA), β-catenin (51067-2-AP, Proteintech, Hubei, China), and GAPDH (60004-AP, Proteintech, Hubei, China). After washing with a tris-buffered saline (TBST) buffer, the membranes were further incubated with a secondary antibody (1:5,000, Abcam, MA, USA) at room temperature for 1 h and then detected by using chemiluminescence reagents (ECL Kit, Biotime, Beijing, China).

### Invasion and Migration Assay

Cell migration, invasion, and luciferase reporter assays were performed according to the methods described in a previous study (30). Detailed information was provided in the Supplementary Methods.

### Luciferase Reporter Assay

Plasmid DNA, either a pmirGLO-WTAP wild type (wt) or pmirGLO-WTAP mutant (mt) (carrying a wt or mt miR-139-5p binding site, respectively), was cotransfected with either a miR-139-5p mimic or an NC mimic into HEK293T cells by using a Lipofectamine-mediated gene transfer. The firefly and Renilla luciferase activities were measured for 48 h after the transfection as described by the manufacturer. The ratio of the luminescence yielded by firefly luciferase to the ratio of the luminescence yielded by Renilla luciferase was calculated as the relative luciferase activity according to a method described in a previous study (29).

### Immunohistochemical Analysis

The immunohistochemical (IHC) staining of paraffin-embedded tissue sections was processed as described in a previous study (31). The primary antibodies used in the present study were as follows: WTAP (60188-1-Ig, Proteintech, Hubei, China), E-cadherin (20874-1-AP, Proteintech, Hubei, China), N-cadherin (22018-1-AP, Proteintech, Hubei, China), Slug (ab106077, Abcam, MA, USA), Snail (ab53519, Abcam, MA USA), and β-catenin (51067-2-AP, Proteintech, Hubei, China).

### HCC Xenograft Model

About 12 male BALB/c nude mice (6–8 weeks old) were purchased from the Shanghai SLAC Animal Center (Shanghai, China). The mice were randomly divided into two groups (sh-WTAP or Ctrl group). SMMC-7721 cells stably transfected with sh-WTAP or ctrl (5 × 10^6^ cells) were subcutaneously injected into the flanks of nude mice. The tumor size was measured every week. The tumor volumes were calculated by using the following equation: volume (mm^3^) = length × width^2^/2. After 5 weeks, the mice were sacrificed, and the tumor weights were measured. The animal experiments were approved by the Animal Care and Use Committee of Zhengzhou University. All the experimental procedures involving animals were performed strictly in accordance with the Guide for the Care and Use of Laboratory Animals as described in a previous study (32).

### Statistical Analysis

All the experiments were repeated thrice. The data are presented as the mean ± SE. Graphs were generated by using the GraphPad Prism software (version 7.0, La Jolla, CA, USA). ANOVA and Tukey's tests were carried out for a multiple-group comparison. A value of *p* < 0.05 was considered as statistically significant.

## Results

### A High Level of WTAP Protein Expression Is Significantly Correlated With a Poor Prognosis in Patients With HCC

**Figure 1:**
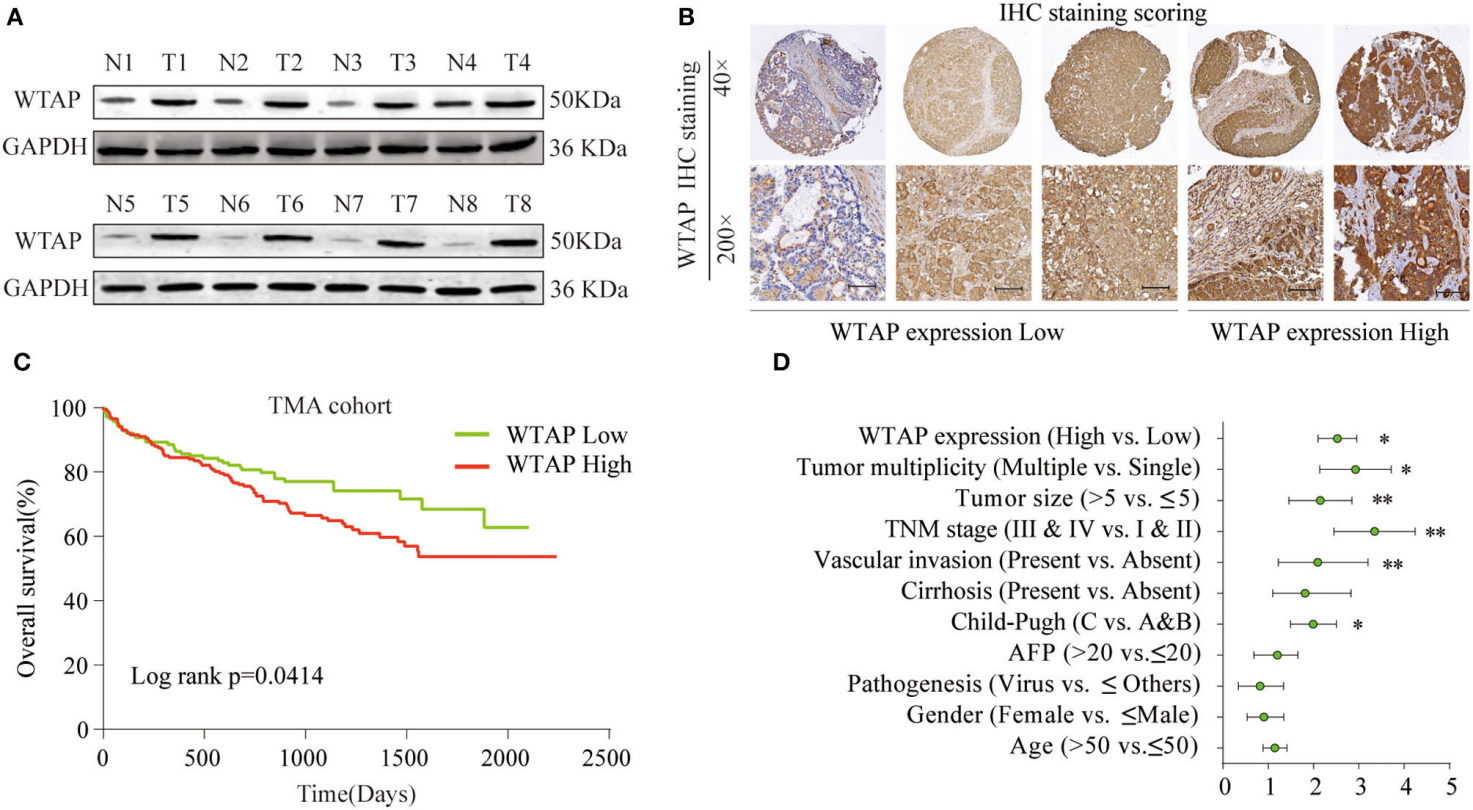
High Wilms' tumor 1-associating protein (WTAP) expression is significantly correlated with a poor prognosis in patients with hepatocellular carcinoma (HCC). **(A)** WTAP expression in eight-paired HCC tissues (T) and the adjacent non-cancer tissues (N) was analyzed by using a western blot. **(B)** Representative immunohistochemical (IHC) staining of WTAP in HCC tissues and the relative scores of the WTAP expression based on the WTAP staining intensity. **(C)** The Kaplan–Meier analysis was used to analyze a correlation between the WTAP expression and the overall survival (OS) of patients with HCC. **(D)** Univariate analysis of the association between the WTAP expression and different clinicopathological features. *p < 0.05, **p < 0.01 based on the non-parametric test.

To determine the WTAP expression profile in HCC tissues, eight-paired surgical HCC and para-carcinoma tissues were analyzed by a western blot. As shown in Figure 1A, the expression of WTAP in liver cancer tissues is significantly higher than that in the adjacent tissues. We further performed an IHC analysis of the WTAP expression using a tissue microarray (TMA) containing 396 HCC and para-carcinoma tissues. Based on the relative intensity of the IHC staining, the WTAP expression was classified into five subgroups with different scores (Figure 1B). Similarly, the IHC staining further confirmed an upregulated WTAP expression in HCC tissues (Supplementary Figure 1A). Moreover, the WTAP expression was positively correlated with the advanced stages of Tumor-Node-Metastasis (TNM) and high rates of vascular invasion (Supplementary Figure 1B). Furthermore, an increase in the WTAP expression was significantly associated with a poor overall survival (OS) (Figure 1C). A multivariate Cox regression analysis indicated that the WTAP expression was an independent prognostic factor, in addition to the TNM stage and vascular invasion, for the OS in patients (Figure 1D). These findings suggested that WTAP was an unfavorable prognostic factor in the HCC progression.

### Correlation Between the mRNA Levels of WTAP and the Clinicopathological Features of Patients With HCC

**Figure 2:**
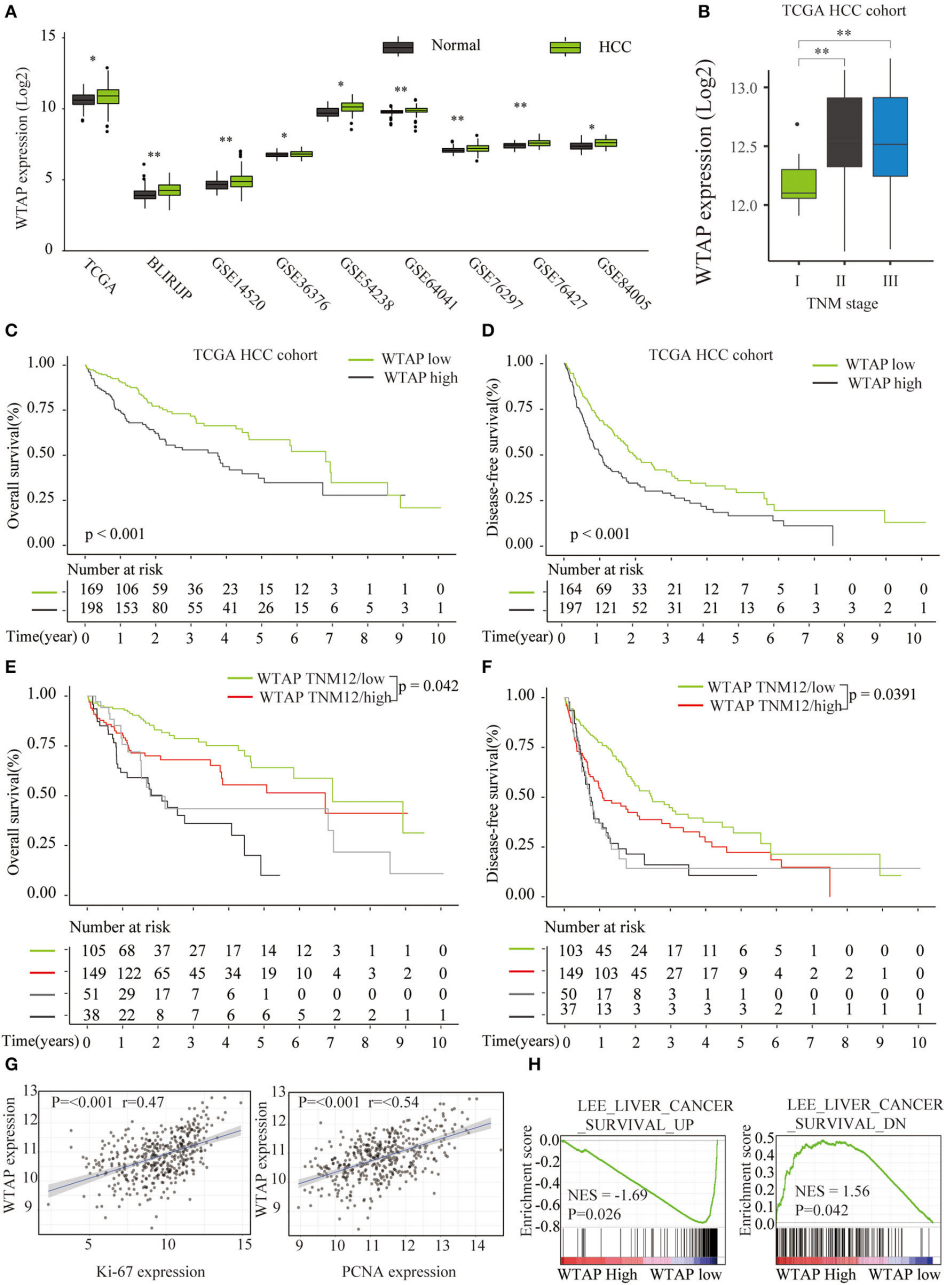
Bioinformatics analysis of a correlation between an elevated WTAP expression and a poor survival of the patients with HCC in public databases. **(A)** Quantification of WTAP messenger RNA (mRNA) expression levels in patients with HCC in The Cancer Genome Atlas (TCGA) and Gene Expression Omnibus (GEO) data sets. **(B)** WTAP mRNA expression levels in HCC tissues with different Tumor-Node-Metastasis (TNM) stages based on a TCGA HCC cohort analysis. **(C,D)** A prognostic significance of the WTAP expression in patients with HCC was analyzed in the TCGA (*n* = 367) database. **(E,F)** A prognostic significance of the WTAP expression in patients with HCC at different TNM stages was analyzed in the TCGA (*n* = 367) database. **(G)** Pearson correlation analysis of WTAP expression levels with the Ki-67 or proliferating cell nuclear antigen (PCNA) expression in TCGA HCC data sets. **(H)** Gene set enrichment analysis (GSEA) of a correlation between the WTAP expression and gene signatures of the survival in HCC. *p < 0.05, **p < 0.01 based on the non-parametric test.

We further analyzed the prognostic role of WTAP in HCC based on the publicly available databases (GEO and TCGA). Nine HCC microarray data sets were analyzed, and the results showed a markedly elevated WTAP expression in HCC in all the data sets (Figure 2A). Moreover, a higher WTAP expression was significantly correlated with the advanced stages of TNM (Figure 2B). A survival analysis *via* Kaplan–Meier and log-rank tests showed that patients with a high WTAP expression had a worse prognosis than those with a low WTAP expression in a TCGA HCC cohort (Figures 2C,D), and this was especially true of the early stage (TNM I and II) patients (Figures 2E,F). In addition, the expression of WTAP was positively correlated with the expression of the cancer proliferation markers Ki-67 and proliferating cell nuclear antigen (PCNA) in the HCC samples (Figure 2G). Furthermore, a gene set enrichment analysis (GSEA) revealed a positive relationship between an increased WTAP expression and the gene signatures associated with a poor survival in HCC (Figure 2H). These results further confirmed that WTAP was upregulated in HCC and correlated with an unfavorable prognosis.

### Knockdown of WTAP Inhibits the Proliferation and Invasion of HCC Cells *in vitro*

**Figure 3:**
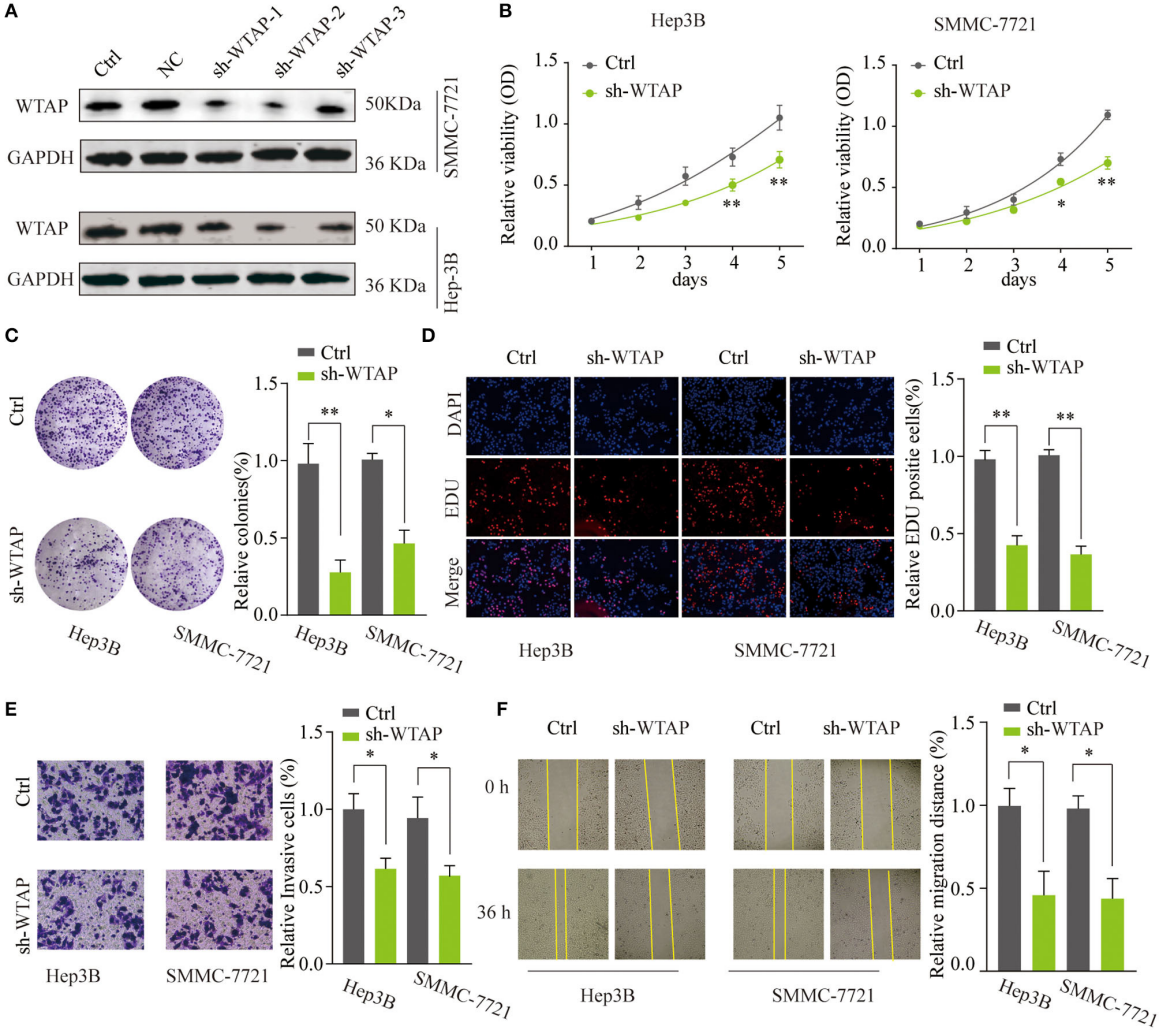
Knockdown of WTAP inhibits the proliferation and invasion of *in vitro* HCC cells. **(A)** SMMC-7721 or Hep3B cells were transfected with different short hairpin RNA (shRNAs) targeting WTAP or control shRNA. WTAP protein expression was analyzed by a western blot 48 h later. GAPDH was used as a loading control. Hep3B or SMMC-7721 cells were transfected with sh-WTAP or control shRNA. **(B–D)** Effects of the knockdown of WTAP on the cell proliferation were determined by a cell counting kit-8 (CCK-8) assay **(B)**, a colony formation assay **(C)**, and the 5-Ethynyl-2′-deoxyuridine (EdU) staining assay **(D)**. Data were represented as the mean ± SD. **(E,F)** The invasion and migration abilities of HCC cells were assessed by using the Transwell invasion **(E)** and wound healing assays **(F)**. *p < 0.05, **p < 0.01.

To investigate the functional role of WTAP in the HCC tumorigenesis, SMMC-7721 or Hep3B cells were transfected with shRNAs targeting WTAP (sh-WTAP-1/2/3) or a NC. The transfection efficiency was examined, and the most efficient shRNA (sh-WTAP-2) was chosen for further experiments (Figure 3A). The CCK-8 assay and colony forming assay indicated that the silencing of WTAP significantly inhibited the proliferation of SMMC-7721 or Hep3B cells (Figures 3B,C). The EdU staining assay revealed a decreased rate of the DNA synthesis after the knockdown of WTAP (Figure 3D). To further investigate the role of WTAP, Transwell invasion and wound-healing assays were conducted. As shown in Figures 3E,F, the knockdown of WTAP markedly attenuated invasion and migration abilities of HCC cells. Taken together, these findings suggested that the loss of WTAP function impaired the aggressiveness of a HCC cell.

### Knockdown of WTAP Suppresses the HCC Tumorigenesis *in vivo*

**Figure 4:**
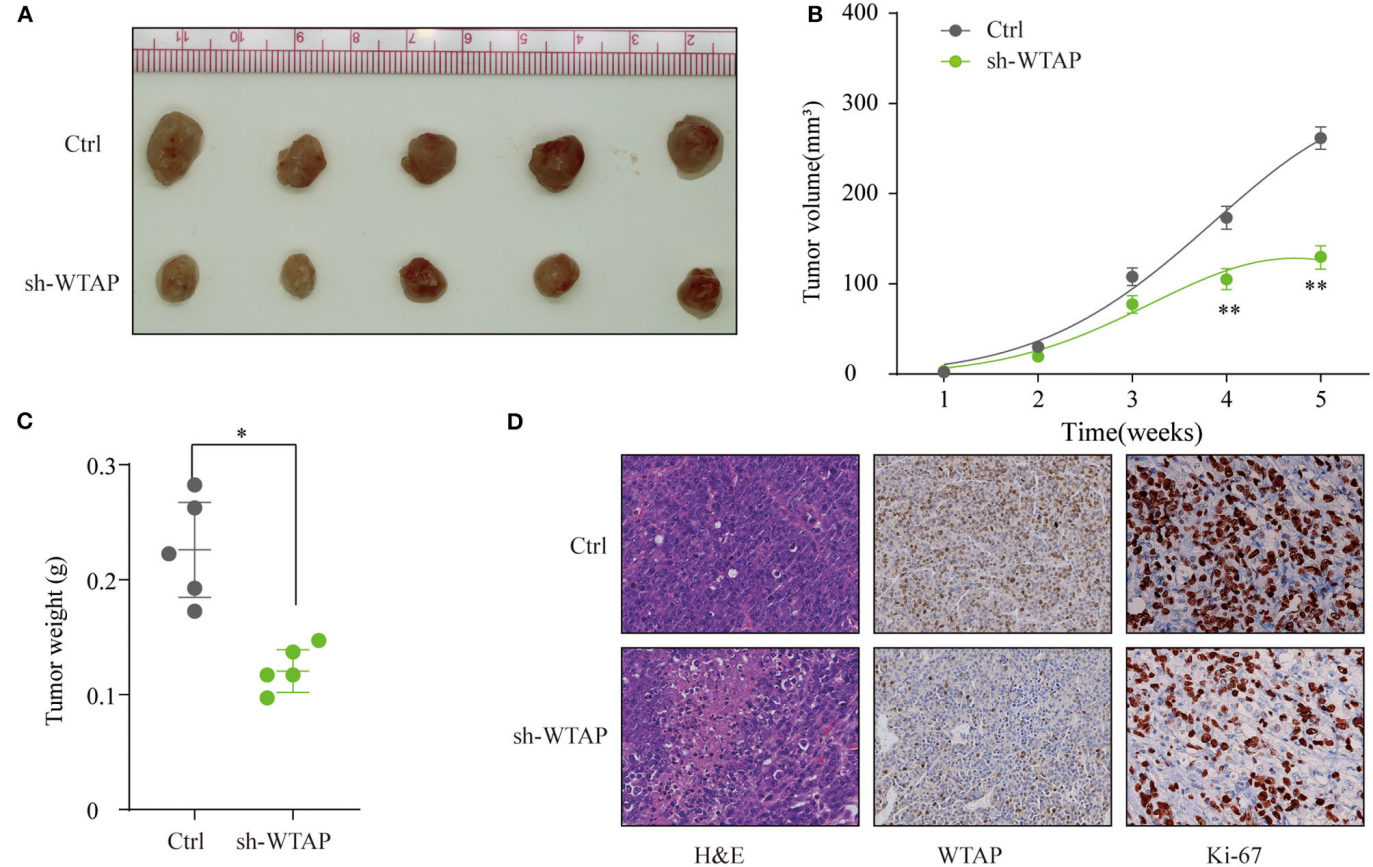
Knockdown of WTAP suppresses the HCC tumorigenesis *in vivo* SMMC-7721 cells stably transfected with a sh-WTAP or negative control (NC) were subcutaneously implanted into nude mice, and the tumor growth was monitored. **(A)** Representative photos of nude mice and HCC tumor tissues from the control (Ctrl) or sh-WTAP group at week 5. **(B)** Tumor volume was monitored and calculated at the indicated time points. **(C)** Tumor weight of tumors from the Ctrl or sh-WTAP group were analyzed; n = 5 per group. **(D)** Representative IHC staining and the expression levels of WTAP or Ki-67 in the tumor tissues from the Ctrl or sh-WTAP group. *p < 0.05, **p < 0.01.

To investigate the oncogenetic role of WTAP *in vivo*, SMMC-7721 cells with the knockdown of stable WTAP or NC cells were subcutaneously implanted into nude mice. As shown in Figure 4A, the knockdown of WTAP significantly delayed the tumor growth and dramatically decreased the tumor volume (Figure 4B). In addition, the tumor weights in the WTAP stable knockdown group were markedly lower than those in the control group (Figure 4C). Furthermore, the IHC staining revealed a lower expression of WTAP and fewer Ki-67-positive cells in the sh-WTAP-transfected tumors (Figure 4D). Taken together, these results further demonstrated that the silencing of WTAP markedly inhibited the HCC tumorigenesis *in vivo*.

### WTAP Promotes EMT in HCC

**Figure 5:**
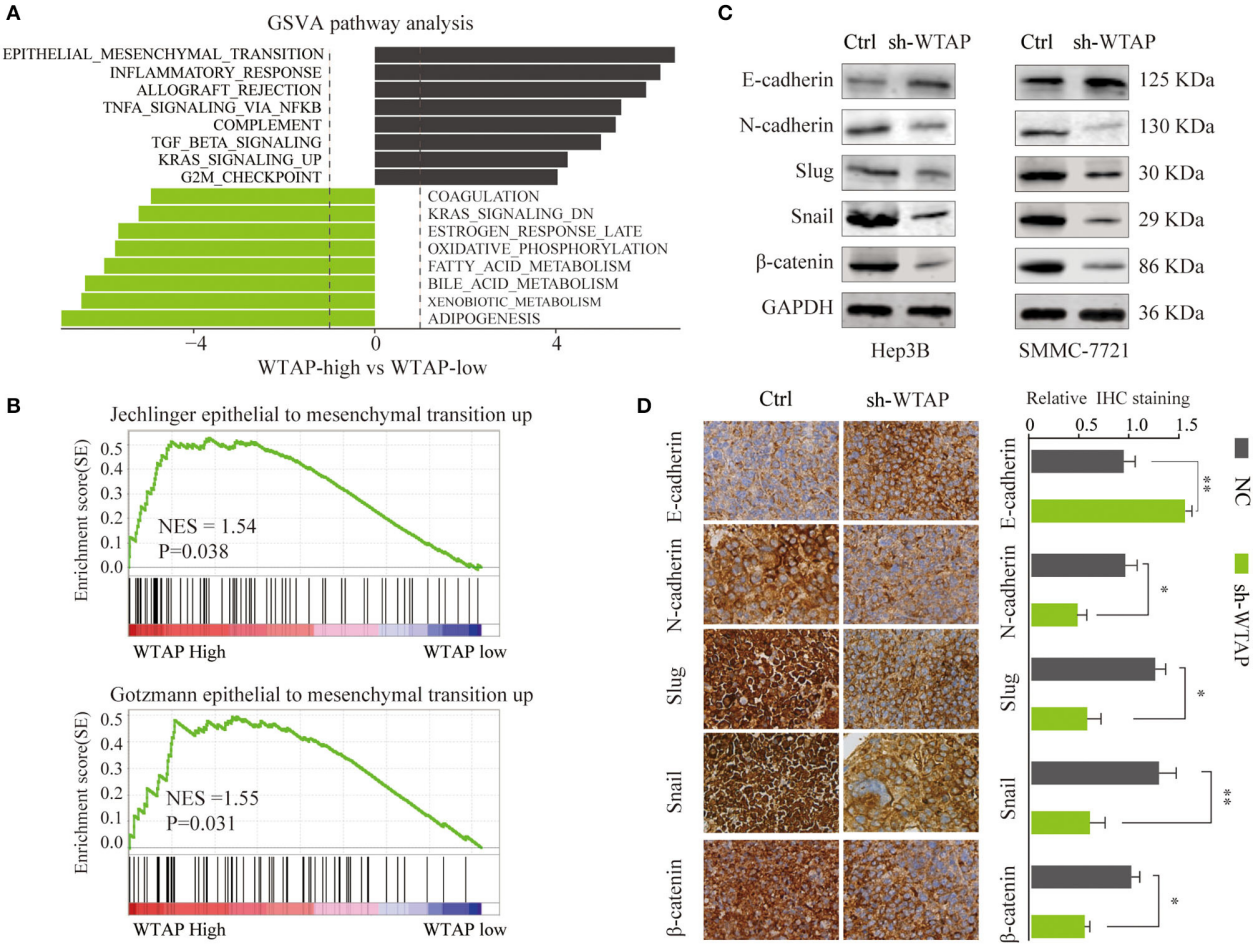
WTAP promotes the epithelial–mesenchymal transition (EMT) in HCC. **(A)** Gene set variation analysis (GSVA) of the signaling pathways in tumors with a high or low WTAP expression in the TCGA HCC database. **(B)** GSEA of a correlation between the WTAP expression and EMT-related gene signatures in HCC. **(C)** A western blot analysis of the protein levels of EMT-related proteins in Hep3B or SMMC-7721 cells transfected with sh-WTAP or controls. GAPDH was used as a loading control. **(D)** IHC staining of E-cadherin, N-cadherin, Slug, Snail, and β-catenin in the tumor tissues from the NC or sh-WTAP group. Scale bar, 100 μm. *p < 0.05, **p < 0.01.

To explore the molecular mechanisms using which WTAP promotes the proliferation, migration, and invasion in HCC cells, we conducted a comprehensive bioinformatics analysis. A gene set variation analysis (GSVA) showed that a high level of the WTAP expression was associated with an activation of the EMT pathway (Figure 5A). GSEA further confirmed a significant positive correlation between a high WTAP expression and EMT-related gene signatures in a consistent manner (Figure 5B). To further determine whether WTAP could accelerate the HCC progression by promoting EMT, we determined the expression levels of EMT-related proteins in Hep3B or SMMC-7721 cells transfected with a sh-WTAP or control. As shown in Figure 5C, in comparison with the NCs, the knockdown of WTAP led to a marked inhibition of several mesenchymal factors, including Snail, N-cadherin, and Slug, and an increased E-cadherin expression. The IHC staining of the tumor tissues from the sh-WTAP or control group showed similar trends of the EMT-related protein expression in a consistent manner (Figure 5D). These findings indicated that WTAP might contribute to the HCC progression by promoting EMT.

### WTAP Is a Direct Binding Target of miR-139-5p

**Figure 6:**
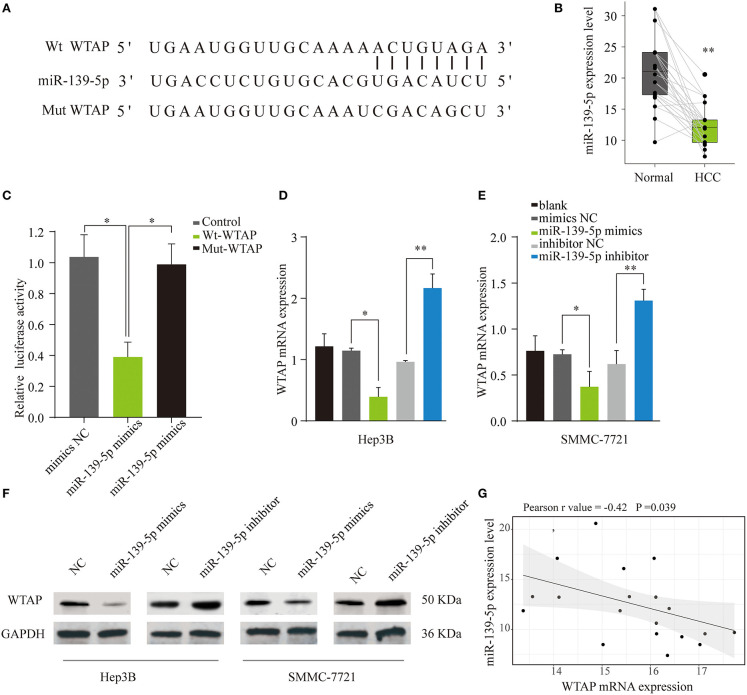
WTAP is a direct binding target of miR-139-5p. **(A)** Predicted target site of miR-139-5p on the 3′ untranslated region (3'-UTR) of WTAP and the mutated WTAP 3′-UTR sequences. **(B)** Quantification of the miR-139-5p expression levels in HCC and normal tissues through quantitative PCR (qPCR) (*n* = 22). **(C)** HEK293 cells were transfected with a reporter vector containing wild type (wt) or mutant (mut) 3′-UTR of WTAP, together with miR-139-5p mimics or negative control. The relative luciferase activity was assessed for 48 h later. The mRNA **(D,E)** and protein **(F)** levels of WTAP in Hep3B or SMMC-7721 cells transfected with miR-139-5p mimics, inhibitor, and negative control were analyzed by qPCR and western blot. **(G)** Pearson correlation analysis of the relationship between the WTAP expression and miR-139-5p expression in HCC tissues. *p < 0.05, **p < 0.01.

Given the crucial roles of miRNAs in the cancer progression, we further explored whether miRNAs were involved in the WTAP dysregulation in HCC (33). An online bioinformatics tool TargetScan was used to search for miRNAs that could regulate WTAP. Intriguingly, miR-139-5p, as a tumor suppressor, was identified to have a seed sequence in the 3′-UTR of WTAP mRNA (Figure 6A). In accordance with the previous reports, miR-139-5p was significantly downregulated in HCC (25) (Figure 6B). In addition, a dual luciferase reporter assay showed that the overexpression of miR-139-5p could inhibit the luciferase activity in the HEK293 cells transfected with the reporter plasmid containing the WT WTAP 3′-UTR, while no significant suppression was found in the HEK293 cells transfected with the plasmid containing the mt WTAP 3′-UTR (Figure 6C). Moreover, the WTAP mRNA and protein levels in SMMC-7721 or Hep3B cells were dramatically decreased by the miR-139-5p overexpression and were increased by the transfection with an miR-139-5p inhibitor (Figures 6D–F). Furthermore, the WTAP expression was negatively correlated with the miR-139-5p expression in the HCC samples (Figure 6G). Together, these data validated that miR-139-5p directly regulated the WTAP expression by targeting 3′-UTR of WTAP.

### The miR-139-5p/WTAP Axis Affects the Proliferation and Invasion of *in vitro* HCC Cells

**Figure 7:**
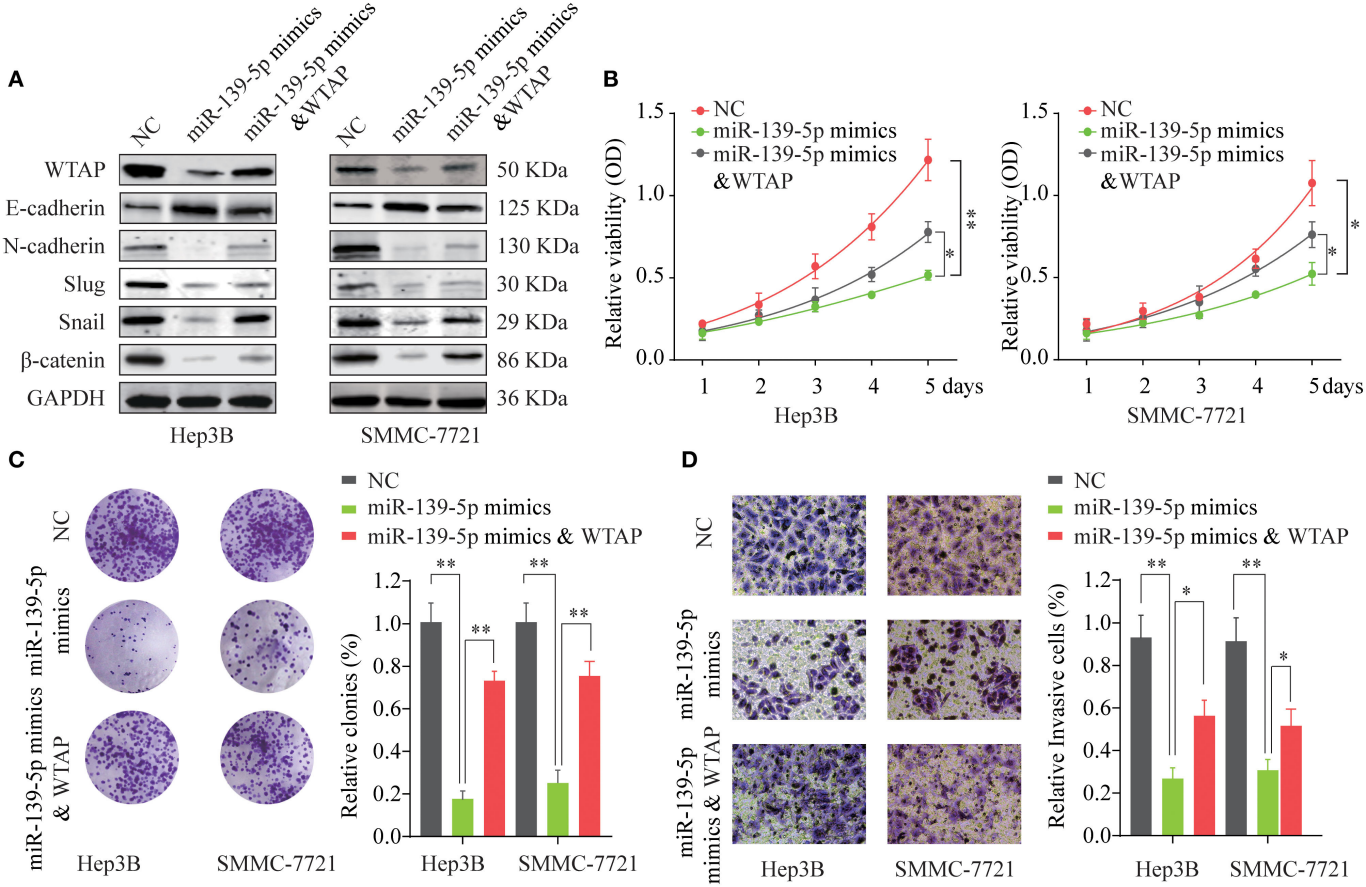
Overexpression of miR-139-5p inhibits the cell proliferation and invasion by targeting WTAP. Hep3B or SMMC-7721 cells were transfected with NC, miR-139-5p mimics or miR-139-5p mimics, and the WTAP overexpression plasmid. **(A)** The expression levels of E-cadherin, N-cadherin, Slug, Snail, and β-catenin were analyzed by western blot 48 h later. GAPDH was used as a loading control. **(B,C)** Cell proliferation was determined by the CCK-8 cell proliferation assay **(B)** and the colony formation assay **(C)**. **(D)** The cell invasion capability of Hep3B or SMMC-7721 cells in the different groups was analyzed by the Transwell assay. *p < 0.05, **p < 0.01.

A previous study indicated that miR-139-5p could compromise a malignant biological behavior in multiple human cancers, including HCC (25). To investigate whether miR-139-5p functions as a tumor suppressor by targeting the WTAP in HCC, rescue experiments were performed. As indicated in Figure 7A, in comparison with the control, the miR-139-5p overexpression decreased the expression of EMT-related proteins, such as Snail, N-cadherin, Slug, and β-catenin, and increased the expression of E-cadherin in Hep3B and SMMC-7721 cells. Cotransfection of the WTAP overexpression vector together with the miR-139-5p mimics reversed the suppressive effects of the miR-139-5p overexpression on EMT-related proteins (Figure 7A). The overexpression of WTAP restored the cell proliferation ability inhibited by the miR-139-5p overexpression as demonstrated by CCK-8 and colony formation assays in a functional manner (Figures 7B,C). Furthermore, we found that the overexpression of WTAP relieved the inhibition of invasion caused by the miR-139-5p overexpression (Figure 7D, Supplementary Figure 2). These data suggested that miR-139-5p inhibited the aggressiveness of HCC cells by regulating the WTAP expression.

### WTAP/miR-139-5p Expression Is Associated With the Prognosis in Patients With HCC

**Figure 8:**
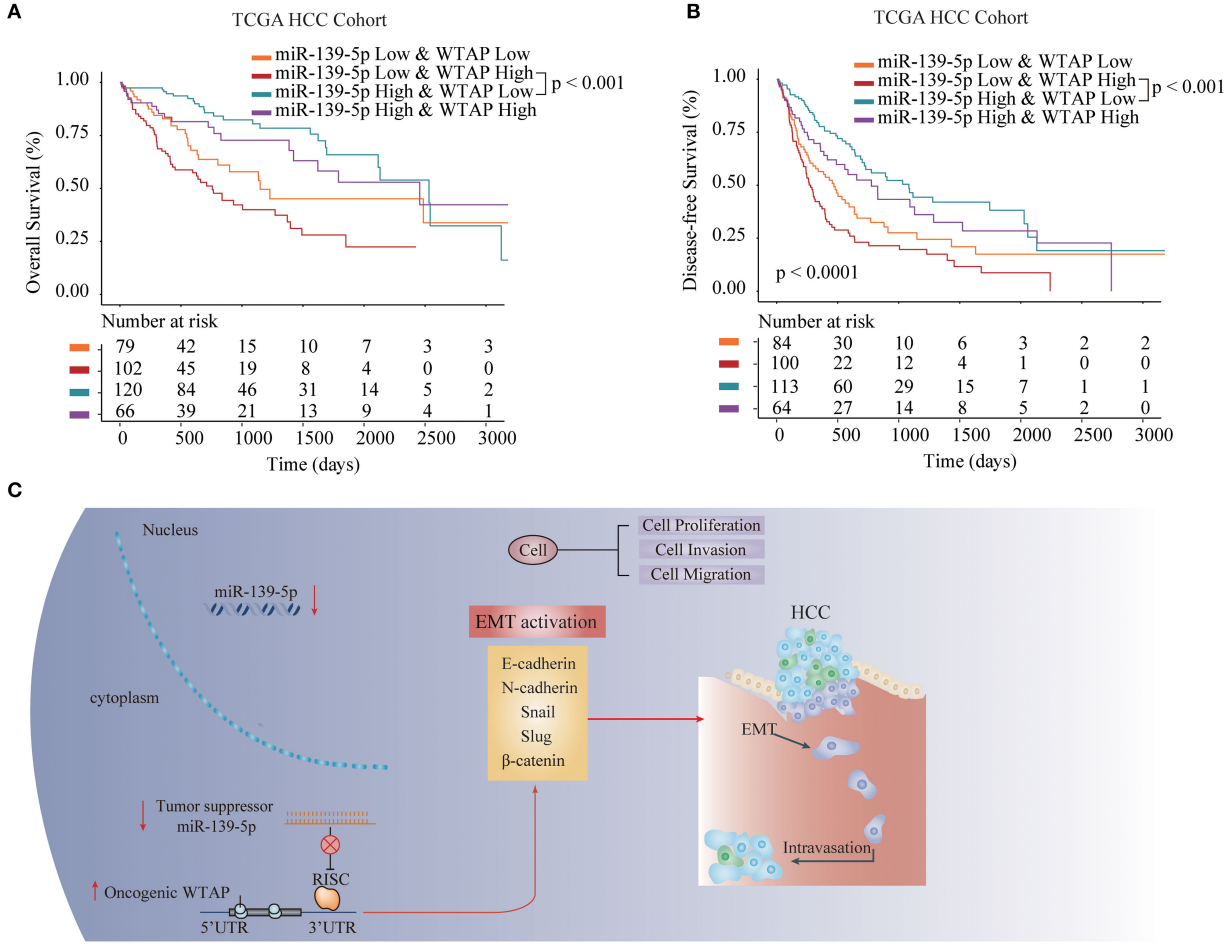
WTAP/miR-139-5p expression is associated with the prognosis in patients with HCC. The Kaplan–Meier analysis of the OS **(A)** and disease-free survival (DFS) **(B)** in patients with HCC having the different expression levels of miR-139-5p and WTAP. **(C)** The proposed working model of the miR-139-5p/WTAP axis in HCC. miR-139-5p functions as a tumor suppressor through inhibiting the WTAP expression in HCC. miR-139-5p/WTAP axis plays a substantial role in inhibiting the tumorigenesis and reversing the metastasis of HCC through the EMT process.

To investigate the prognostic role of the miR-139-5p/WTAP axis in patients with HCC, we divided a TCGA HCC cohort into four groups, including the miR-139-5p^low^WTAP^low^, miR-139-5p^low^WTAP^high^, miR-139-5p^high^WTAP^low^, and miR-139-5p^high^WTAP^high^ group. We found that patients with HCC having miR-139-5p^low^WTAP^high^ had a worse OS and disease-free survival (DFS) rates than patients with HCC having miR-139-5p^high^WTAP^low^ (Figures 8A,B). Collectively, these results further indicated that miR-139-5p negatively regulated the WTAP expression and inhibited the HCC progression *via* an EMT signaling pathway (Figure 8C).

## Discussion

A growing body of evidence suggests that WTAP plays crucial roles in the tumorigenesis and progression of a variety of malignant tumors (12, 34). Tian et al. showed that a high WTAP expression was significantly associated with a poor prognosis in patients with pancreatic cancer (16). Xi et al. (18) reported that the WTAP expression was elevated in glioma tissues and closely correlated with a worse survival in patients with glioma. Similar results were also observed in the bladder cancer, renal cell carcinoma, and HCC (17, 21, 35). In agreement with the above findings, we first revealed that WTAP was significantly upregulated in HCC tissues, and a high WTAP expression was associated with the advanced stages of TNM, a vascular invasion, and a worse prognosis. Moreover, multivariate analyses indicated that WTAP could be a novel and an effective biomarker for the risk prediction in HCC. These findings prompted us to investigate whether a high WTAP overexpression is a potential biomarker for the poor prognosis in patients with HCC.

Next we explored the biological function of WTAP in HCC. We found that the silencing of WTAP significantly impaired the proliferation, migration, and invasion of *in vitro* HCC cells. Moreover, the knockdown of WTAP markedly suppressed the development of HCC tumor *in vivo*. These findings indicated that WTAP might function as an oncogenic protein in the development of HCC. Consistent with our study, Bansal et al. (14) found that the knockdown of WTAP could significantly inhibit the cell proliferation and arrest a differentiation of leukemia cells. Xi et al. confirmed the role of promoting the WTAP proliferation and metastasis in glioblastoma cells (18). In addition, WTAP could facilitate cell proliferation and invasion abilities of cholangiocarcinoma and renal cell carcinoma cells (17). These findings confirmed that WTAP could act as an oncogene in the progression of HCC and might be a promising therapeutic target. However, the underlying mechanism of WTAP involved in the HCC tumorigenicity remains elusive.

Previous studies revealed that WTAP contributed to the cancer progression by increasing the expression of several oncogenes, such as MMP-7, MMP28, cathepsin H, and Muc1 (36). WTAP could also activate an epidermal growth factor signaling (15). Moreover, the functional effect of WTAP was proven to be mediated by regulating a mechanistic target of rapamycin (mTOR) signaling pathway (14). A previous study confirmed that WTAP regulated the cyclin dependent kinase 2 (CDK2) mRNA stability in renal cell carcinoma (17), while Chen et al. (21) reported that WTAP promoted the progression of HCC in a m6A-dependent manner. In the present study, we performed a comprehensive bioinformatics analysis and found that a high WTAP expression was markedly correlated with the gene signatures of EMT signaling. The knockdown of WTAP significantly altered the expression levels of EMT markers in HCC cells. Furthermore, the change in EMT-related proteins was also verified in xenograft tumor tissues by using an IHC analysis. Taken together, our findings provide a novel insight into the oncogenic role of WTAP in the development of HCC. However, further studies are needed to illustrate the molecular mechanism using which WTAP promotes EMT signaling.

Additionally, bioinformatics analysis and validation assays were used to identify the miRNAs that regulate WTAP. MiR-139-5p was identified to have a putative binding sequence in the 3′-UTR of WTAP, and miR-139-5p negatively regulated the WTAP expression in HCC cells. MiR-139-5p has been reported to be associated with the progression of several solid tumors, including HCC (25). We further demonstrated that the WTAP overexpression could partially reverse the inhibitory function of miR-139-5p on the cell malignant biological behavior. Moreover, the WTAP overexpression reversed the suppressive effects of the miR-139-5p overexpression on EMT pathway-related proteins. Moreover, we found that patients with HCC having miR-139-5p^low^WTAP^high^ had worse OS and DFS rates than patients with HCC having miR-139-5p^high^WTAP^low^. Taken together, the results suggest that the miR-139-5p/WTAP axis plays a pivotal role in the pathogenesis and prognosis of HCC by, at least in part, regulating the EMT process.

## Conclusion

In conclusion, we report that WTAP is an oncogene in HCC, and an elevated WTAP expression is an important prognostic factor in HCC. Moreover, WTAP is shown to be a functional target of miR-139-5p. The miR-139-5p/WTAP axis contributes to the HCC progression mainly by regulating the EMT process, which may promote the development of miR-139-5p/WTAP axis-directed diagnostic and therapeutic strategies for HCC.

## Data Availability Statement

Publicly available datasets were analyzed in this study. This data can be found here: TCGA (The Cancer Genome Atlas) and GEO (Gene Expression Omnibus) database.

## Ethics Statement

The studies involving human participants were reviewed and approved by the human ethic committee of The First Affiliated Hospital of Zhengzhou University. The patients/participants provided their written informed consent to participate in this study. The animal study was reviewed and approved by the human ethic committee of The First Affiliated Hospital of Zhengzhou University. Written informed consent was obtained from the owners for the participation of their animals in this study.

## Author Contributions

WL, WS, and ML conceived and designed the study. WL, XG, NZ, YG, LY, GW, BW, and XP performed the experiments. XC, YS, and YZ collected the clinical samples. WL, WS, and ML analyzed the data and wrote the manuscript. All the authors reviewed and approved the manuscript.

## Conflict of Interest

The authors declare that the research was conducted in the absence of any commercial or financial relationships that could be construed as a potential conflict of interest.

## Abbreviations

HCC: hepatocellular carcinoma
OS: overall survival
TMAs: tissue microarrays
qRT-PCR: quantitative real-time PCR
CCK-8: cell counting kit-8
3′-UTR: 3′ untranslated region
GSEA: gene set enrichment analysis
SD: standard deviation.
