# Supplementary material for: miR-139-5p Loss-Mediated WTAP Activation Contributes to Hepatocellular Carcinoma Progression by Promoting the Epithelial to Mesenchymal Transition
Source: Front Oncol. 2021 Apr 15;11:611544. doi: 10.3389/fonc.2021.611544 (PMC8083052; doi:10.3389/fonc.2021.611544)
Supplement: Supplementary file 1 [file Data_Sheet_1.pdf]

Supplementary Methods

Wound healing assay

For the evaluation of cell migration ability, a wound healing assay was performed as described previously. Briefly, the cells were seeded into the six-well plates and subjected to different treatments. When the cells reached about 90% confluence, a wound was scratched with a sterile 20 μl pipette tip. The cell layers were imaged, and the migration ability of the cells was observed at 0 and 36 h after scratching using an Olympus 1X71 camera system. At the indicated time points, the images were taken under a microscope.

5-Ethynyl-2'-deoxyuridine (EdU) incorporation assay

The EdU assay was performed with a Cell-Light EdU DNA Cell Proliferation Kit (RiboBio, China). Cells (1×10^4) were seeded in each well of 96-well plates. After incubation with 50 mM EdU for 2 h, the cells were fixed in 4% paraformaldehyde and stained with Apollo Dye Solution. DAPI was used to stain the nucleic acid within the cells. Images were obtained with an Olympus microscope (Olympus, Japan), and the number of EdU-positive cells was counted.

Colony formation assay

For the colony formation assays, the cells were trypsinized, and 1×10^3 cells were plated in 6-well plates and incubated for 14 days. Colonies were dyed with dyeing solution containing 0.1% crystal violet and 20% methanol. Cell colonies were then counted and analyzed.

Invasion and migration assay

The capacity for cell migration and invasion was evaluated by using Transwell chambers (Corning, USA). After pretreatment, 4×10^4 cells suspended in 200 μL of serum-free medium were seeded into the upper chamber of the Transwell system, and medium supplemented with 10% FBS was added to the lower chamber. For invasion

assays, cells were seeded in pre-coated Matrigel Transwell insert chambers. After incubation for 24 h, cells remaining on the top surface were removed, and cells migrated to the lower surface of the membrane were fixed and stained with 0.1% crystal violet.
