# Supplementary material for: miR-139-5p Loss-Mediated WTAP Activation Contributes to Hepatocellular Carcinoma Progression by Promoting the Epithelial to Mesenchymal Transition
Source: Front Oncol. 2021 Apr 15;11:611544. doi: 10.3389/fonc.2021.611544 (PMC8083052; doi:10.3389/fonc.2021.611544)
Supplement: Supplementary file 2 [file Image_1.pdf]

Supplementary Figure S1

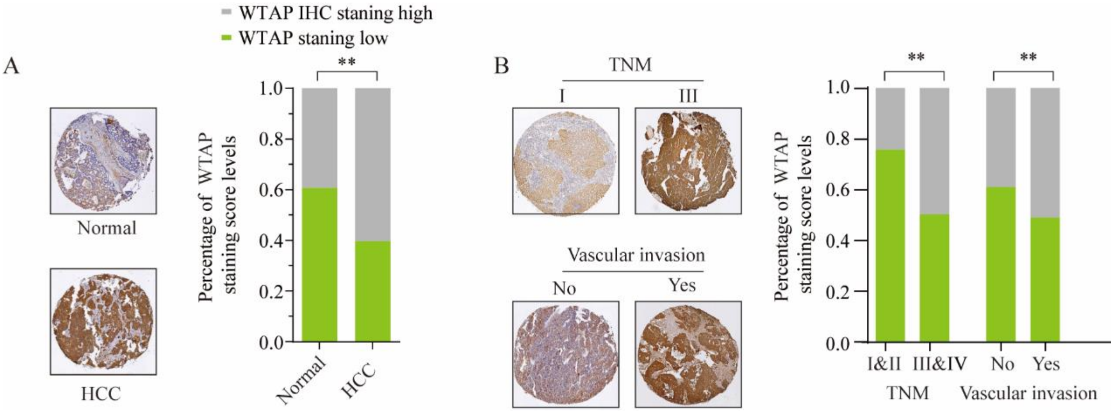

Figure S1 (A) Representative IHC staining of WTAP expression in HCC and normal tissues (left) and the distribution of WTAP staining scores in HCC tissues and adjacent noncancer tissues. (B) Representative images of WTAP IHC staining and the distribution of WTAP IHC staining scores in HCC tissues with different TNM stages, with or without vascular invasion. $^{**}p < 0.01$.
