# Supplementary material for: miR-139-5p Loss-Mediated WTAP Activation Contributes to Hepatocellular Carcinoma Progression by Promoting the Epithelial to Mesenchymal Transition
Source: Front Oncol. 2021 Apr 15;11:611544. doi: 10.3389/fonc.2021.611544 (PMC8083052; doi:10.3389/fonc.2021.611544)
Supplement: Supplementary file 3 [file Image_2.pdf]

Supplementary Figure S2

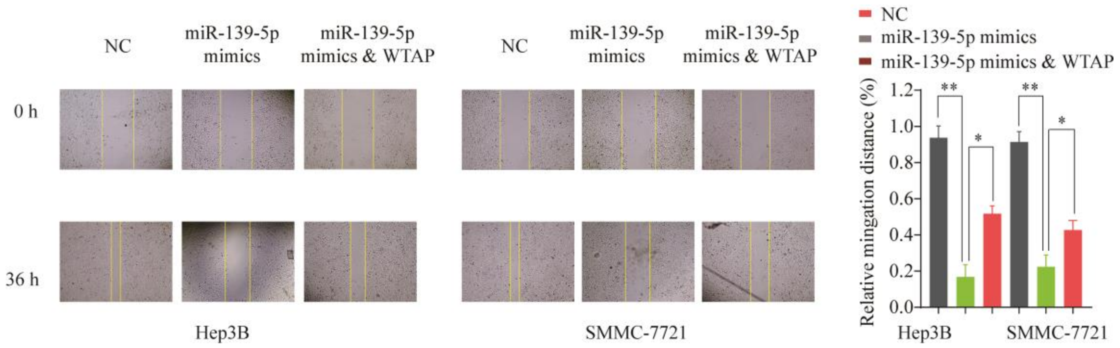

Figure S2 Hep 3B or SMMC-7721 cells were transfected with NC, miR-139-5p mimics or miR-139-5p mimics & WTAP overexpression plasmid. Cell migration capability of Hep 3B or SMMC-7721 cells in different groups was analyzed by transwell assay. *$p < 0.05$, **$p < 0.01$.
