# Supplementary material for: miR-139-5p Loss-Mediated WTAP Activation Contributes to Hepatocellular Carcinoma Progression by Promoting the Epithelial to Mesenchymal Transition
Source: Front Oncol. 2021 Apr 15;11:611544. doi: 10.3389/fonc.2021.611544 (PMC8083052; doi:10.3389/fonc.2021.611544)

SHANGHAI BIOWING BIOTECHNOLOGY Co. LTD

Your Intimate Partner in Bioscience!

Cell Line Authentication Report STR Profiling

Sample Type: Cell Line

Sample from: The First Affiliated Hospital of Zhengzhou University

Testing Method: STR Genotyping

Report Time: May 17, 2017

COMPANY STATEMENT

1. THIS REPORT IS ONLY RESPONSIBLE FOR THE SAMPLES ANALYZED.
2. THE TESTING RESULTS AND THE ORGANIZATION NAME WILL NOT BE USED FOR ADVERTISEMENT, COMMERCIAL EXHIBITIONS, COMMERCIAL PERFORMANCE AND OTHER COMMERCIAL ACTIVITIES.
3. OBJECTIONS SHOULD BE RAISED WITHIN FIFTEEN DAYS AFTER THE RECEIPT OF THIS REPORT.
4. THE PAPER REPORT WITH CONTENT ALTERING, ADDING ARE INVALID.

Testing Company: Shanghai Biowing Applied Biotechnology Co. Ltd

Address: Room 4F, 8 th Buiding,Guiguo Garden,NO.471 Guiping Road,Caohejing Development Zone,Shanghai

Tel: +86-021-33559491

Contact: YiQun Chen

E-mail: biowing@vip.163.com

Cell Line Authentication – STR Profiling Report

Sample code

Table 1. Sample Code

Customer's code	Company Code
Hep3B	20170510-01

Sample Number:1

Sample Type: Cell line

Testing Type: STR

Sample From: The First Affiliated Hospital of Zhengzhou University

Testing Method:

DNA was extracted by a commercial kit from CORNING (AP-EMN-BL-GDNA-250G). The twenty STRs including Amelogenin locus were amplified by six multiplex PCR and separated on ABI 3730XL Genetic Analyzer. The signals were then analyzed by the software GeneMapper.

Data Interpretation:

Cell lines were authenticated using Short Tandem Repeat (STR) analysis as described in 2012 in ANSI Standard (ASN-0002) by the ATCC Standards Development Organization (SDO) and in Capes-Davis et al., Match criteria for human cell line authentication: Where do we draw the line? Int J Cancer.2013;132(11):2510-9.

Test Results:

1. Result

Table 2. Matching information on the cell lines

Sample Code	Multi-allele	Cell line matched	Cell Bank	EV	Percentage
20170510-01	No	HEP-3B	DSMZ	1	9/9

- Multi-allele means some STR contain more than two loci.

2. Sample Description

20170510-01:

A. The STR results showed that no multiple alleles were found in this cell line, and no cross

contamination of human cells was found in the cell line.

B. The DNA of the cell lines found to perfect match the type of cell lines in a cell line retrieval, DSMZ database shows that cells called **HEP-3B, Hep 3B2.1-7 [Hep 3B, Hep-3B, Hep3B].** corresponding to the cell number **CRL-9444 , 93.**

EV	Cell No.	Cell name	Locus names								
			D5S818	D13S317	D7S820	D16S539	VWA	TH01	AM	TPOX	CSF1PO
	Query (Your Cell)		*13, 13*	*12, 14*	*8, 10*	*10, 10*	*17, 17*	*6, 7*	*x, x*	*9, 9*	*8, 8*
1.00(36/36)	93	HEP-3B	13,13	12,14	8,10	10,10	17,17	6,7	X,X	9,9	8,8
1.00(36/36)	HB-8064	Hep 3B2.1-7 [Hep 3B, Hep-3B, Hep3B]	13,13	12,14	8,10	10,10	17,17	6,7	X,X	9,9	8,8

3. Genotyping Result

Table 3. STR and Amelogenin Genotyping Results of Cell line 20170510-01

Loci	Sample information			Cell Bank information		
	Sample name：HEP3B			Cell line name：HEP-3B		
	Allele1	Allele2	Allele3	Allele1	Allele2	Allele3
D5S818	13	13		13	13	
D13S317	12	14		12	14	
D7S820	8	10		8	10	
D16S539	10	10		10	10	
VWA	17	17		17	17	
TH01	6	7		6	7	
AMEL	X	X		X	X	
TPOX	9	9		9	9	
CSF1PO	8	8		8	8	
D12S391	17	17				
FGA	18	18				
D2S1338	21	25				
D21S11	30	31				
D18S51	20	20				
D8S1179	12	12				

D3S1358	15	15	
D6S1043	12	17	
PENTAE	5	16	
D19S433	12.2	14	
PENTAD	12	14	

Others:

1. Genotyping Strategy and Site Distribution

Attached Table. Experimental Strategy and Sites

	**Strategy 1**	**Strategy 2**	**Strategy 3**	**Strategy 4**
1	TH01	TPOX	D3S1358	AMEL
2	D12S391	VWA	D13S317	D5S818
3	D7S820	D8S1179	D6S1043	D2S1338
4	CSF1PO	PENTAD	D16S539	D21S11
5	FGA		D19S433	D18S51
6	PENTAE			

The allele match algorithm compares the 8 core loci plus amelogenin only, even though alleles from all loci will be reported when available.

2. DSMZ tools was used to carry on the cell line comparison, which contains 2455 cell lines STR data from ATCC, DSMZ, JCRB ,ECACC，GNE and RIKEN databases. If the cell is not included in the above cell library, users need to compared with other databases.

Technician:Menglu Shen
Check: Yang Bai
Person in Charge: Yiqun Chen

Issue date: May 17, 2017

Applied Biosystems
GeneMapper 4.0

Cell Authentication 16

Sample File	Sample Name	Panel	OS	SQ
41_A06_XiBaoJianDing-2-0517.fsa	Hep3B	STR Profile 1-dup		

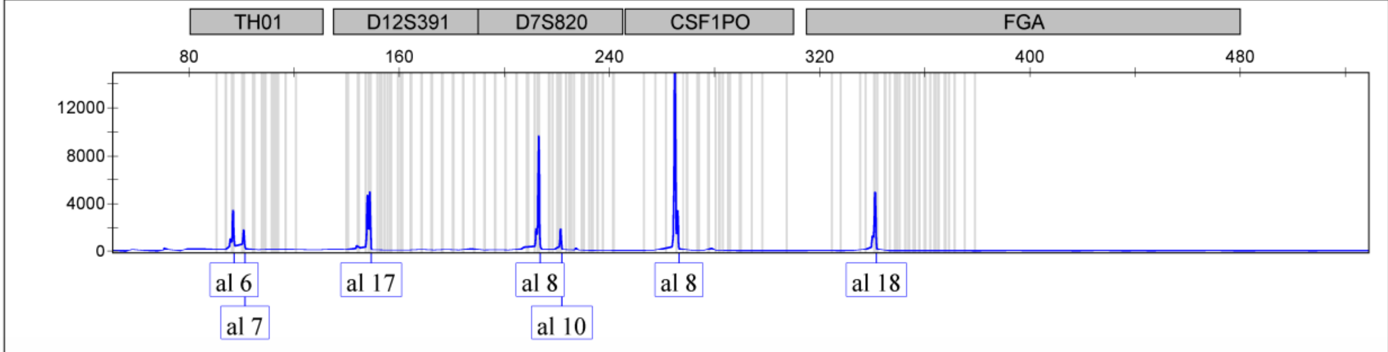

Sample File	Sample Name	Panel	OS	SQ
57_A08_XiBaoJianDing-2-0517.fsa	Hep3B	STR Profile 2-dup		

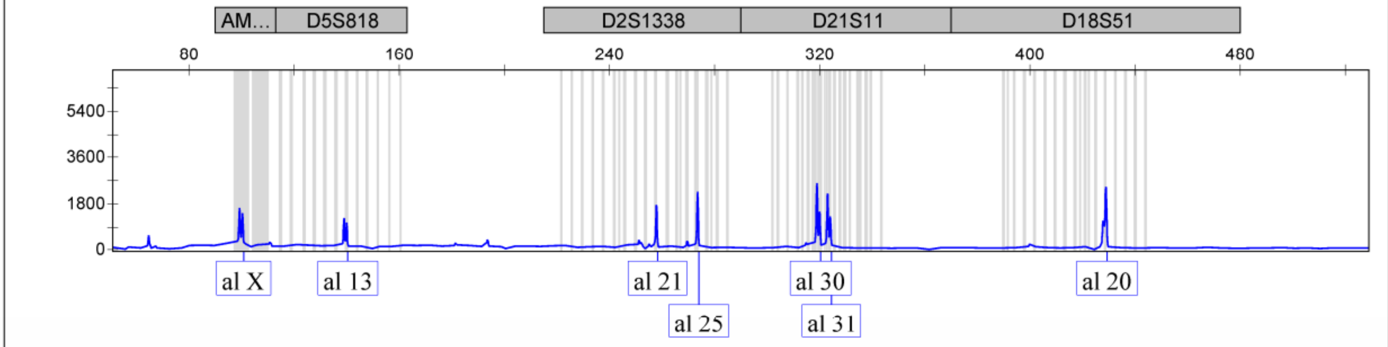

Sample File	Sample Name	Panel	OS	SQ
73_A10_XiBaoJianDing-2-0517.fsa	Hep3B	STR Profile 3-dup		

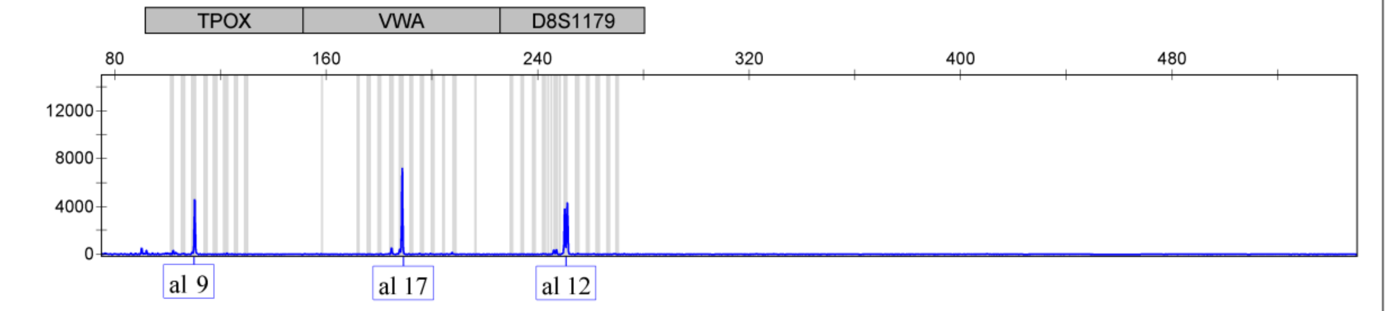

GeneMapper 4.0

Cell Authentication 16

Sample File	Sample Name	Panel	OS	SQ
89_A12_XiBaoJianDing-2-0517.fsa	Hep3B	STR Profile 4-dup		

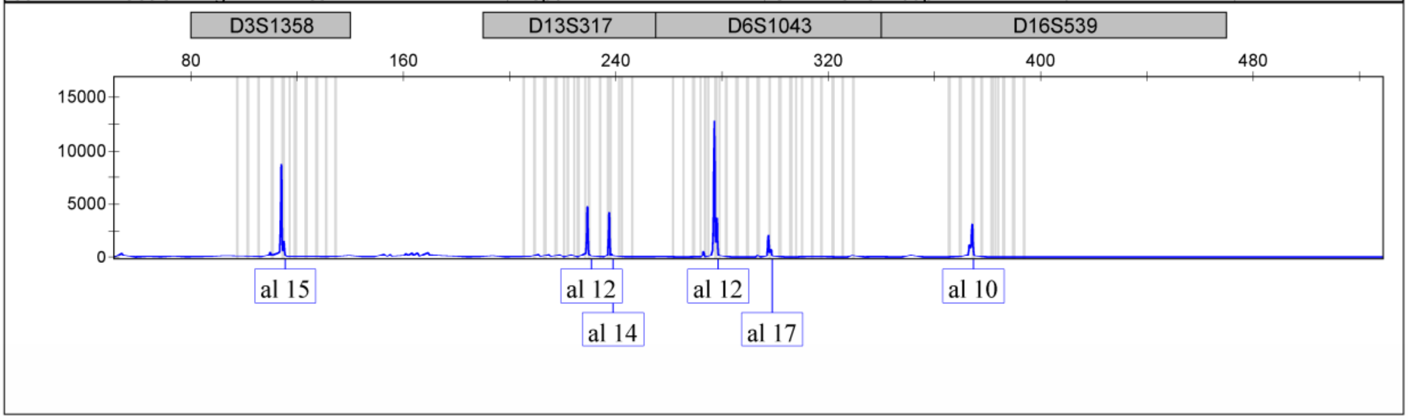

Sample File	Sample Name	Panel	OS	SQ
79_G10_XiBaoJianDing-2-0517.fsa	Hep3B	STR Profile 5-dup		

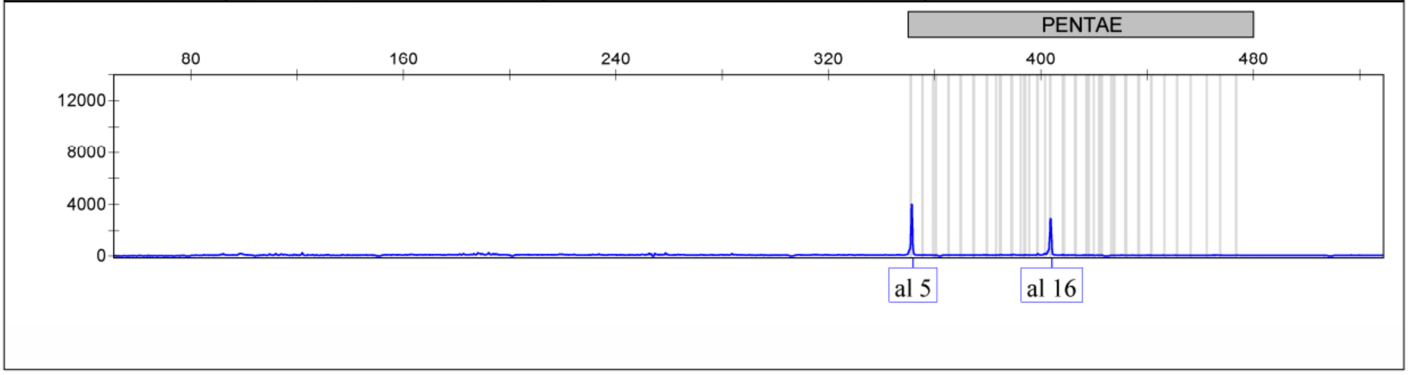

Sample File	Sample Name	Panel	OS	SQ
52_D07_XiBaoJianDing.0517.fsa	Hep3B	STR Profile 6-dup		

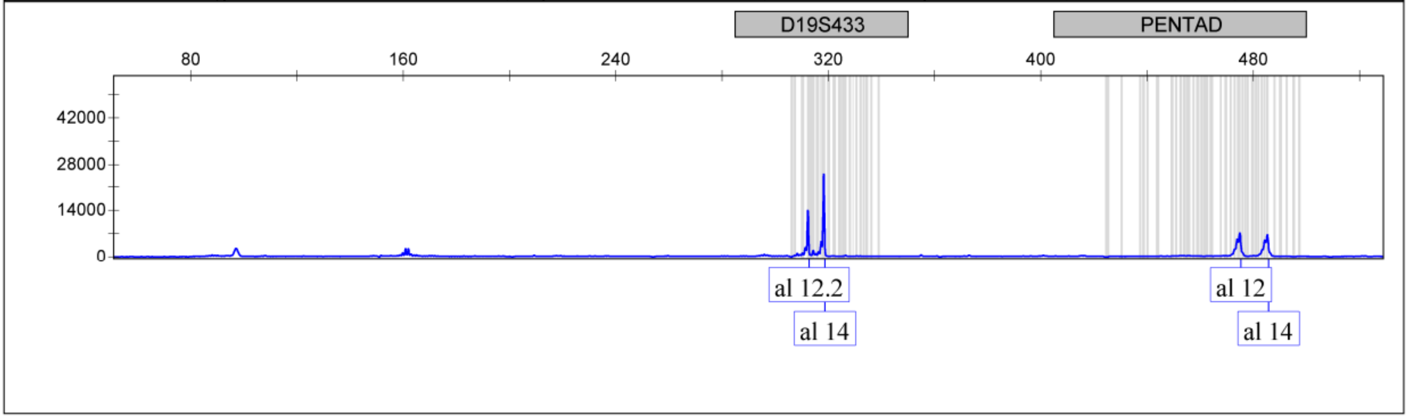

Supplement: Supplementary file 4 [file Data_Sheet_2.pdf]
