# Supplementary material for: miR-139-5p Loss-Mediated WTAP Activation Contributes to Hepatocellular Carcinoma Progression by Promoting the Epithelial to Mesenchymal Transition
Source: Front Oncol. 2021 Apr 15;11:611544. doi: 10.3389/fonc.2021.611544 (PMC8083052; doi:10.3389/fonc.2021.611544)

Cell Line Authentication Report STR Profiling

Sample Type: Cell Line

Sample from: The First Affiliated Hospital of Zhengzhou University

Testing Method: STR Genotyping

Report Time: March 25, 2017

COMPANY STATEMENT

1. THIS REPORT IS ONLY RESPONSIBLE FOR THE SAMPLES ANALYZED.
2. THE TESTING RESULTS AND THE ORGANIZATION NAME WILL NOT BE USED FOR ADVERTISEMENT, COMMERCIAL EXHIBITIONS, COMMERCIAL PERFORMANCE AND OTHER COMMERCIAL ACTIVITIES.
3. OBJECTIONS SHOULD BE RAISED WITHIN FIFTEEN DAYS AFTER THE RECEIPT OF THIS REPORT.
4. THE PAPER REPORT WITH CONTENT ALTERING, ADDING ARE INVALID.

Testing Company: Shanghai Biowing Applied Biotechnology Co. Ltd

Address: Room 4F, 8 th Buiding,Guiguo Garden,NO.471 Guiping Road,Caohejing Development Zone,Shanghai

Tel: +86-021-33559491

Contact: YiQun Chen

E-mail: biowing@vip.163.com

Cell Line Authentication – STR Profiling Report

Sample code

Table 1. Sample Code

Customer's code	Company Code
SMMC-7721	20170320-02

Sample Number:1

Sample Type: Cell line

Testing Type: STR

Sample From: The First Affiliated Hospital of Zhengzhou University

Testing Method:

DNA was extracted by a commercial kit from CORNING (AP-EMN-BL-GDNA-250G). The twenty STRs including Amelogenin locus were amplified by six multiplex PCR and separated on ABI 3730XL Genetic Analyzer. The signals were then analyzed by the software GeneMapper.

Data Interpretation:

Cell lines were authenticated using Short Tandem Repeat (STR) analysis as described in 2012 in ANSI Standard (ASN-0002) by the ATCC Standards Development Organization (SDO) and in Capes-Davis et al., Match criteria for human cell line authentication: Where do we draw the line? Int J Cancer.2013;132(11):2510-9.

Test Results:

1. Result

Table 2. Matching information on the cell lines

Sample Code	Multi-allele	Cell line matched	Cell Bank	EV	Percentage
20170320-02	No	SMMC-7721,etc.	CRC	1	9/9

- Multi-allele means some STR contain more than two loci.

2. Sample Description

20170320-02:

A. The STR results showed that no multiple alleles were found in this cell line, and no cross

contamination of human cells was found in the cell line.

B. The DNA of the cell lines found to basic match the type of cell lines in a cell line retrieval, CRC database shows that cells called **SMMC-7721.** corresponding to the cell number **CCRID; 3111C0001CCC000087.**

匹配结果表															
细胞名	D8S1179	D21S11	D7S820	CSF1PO	D3S1358	TH01	D13S317	D16S539	D2S1338	D19S433	vWA	TPOX	D18S51	Amelogenin	D5S818
你的数据	12,13	27,28	8,12	9,10	15,18	7	12,13.3	9,10	17	13,14	16,18	8,12	13,16	x	11,12
SMMC-7721	12	27,28	12	10	15,18	7	13.3,15.1	9,10	17	13	16,18	12	16	X	11,12

3. Genotyping Result

Table 3. STR and Amelogenin Genotyping Results of Cell line 20170320-02

Loci	Sample information			Cell Bank information		
	Sample name：SMMC-7721			Cell line name：SMMC7721		
	**Allele1**	**Allele2**	**Allele3**	**Allele1**	**Allele2**	**Allele3**
D5S818	11	12		11	12	
D13S317	12	13.3		15.1	13.3	
D7S820	8	12		8	12	
D16S539	9	10		9	10	
VWA	16	18		16	18	
TH01	7	7		7	7	
AMEL	X	X		X	X	
TPOX	8	12		8	12	
CSF1PO	9	10		9	10	
D19S433	13	14		13	14	
FGA	21	21		18	21	
D2S1338	17	17		17	17	
D21S11	27	28		27	28	
D18S51	13	16		13	16	
D8S1179	12	13		12	13	
D3S1358	15	18		15	18	

D6S1043	18	19	
PENTAE	7	17	
D12S391	20	25	
PENTAD	8	15	

Others:

1. Genotyping Strategy and Site Distribution

Attached Table. Experimental Strategy and Sites

	Strategy 1	Strategy 2	Strategy 3	Strategy 4
1	TH01	TPOX	D3S1358	AMEL
2	D12S391	VWA	D13S317	D5S818
3	D7S820	D8S1179	D6S1043	D2S1338
4	CSF1PO	PENTAD	D16S539	D21S11
5	FGA		D19S433	D18S51
6	PENTAE			

The allele match algorithm compares the 8 core loci plus amelogenin only, even though alleles from all loci will be reported when available.

2. DSMZ tools was used to carry on the cell line comparison, which contains 2455 cell lines STR data from ATCC, DSMZ, JCRB ,ECACC，GNE and RIKEN databases. If the cell is not included in the above cell library, users need to compared with other databases.

Technician:Menglu Shen
Check: Yang Bai
Person in Charge: Yiqun Chen

Issue date: March 25, 2017

Sample File	Sample Name	Panel	OS	SQ
28_D04_XiBaoJianDing(0705+0702)-1-0707.fsa	SMMC-7721	STR Profile 1-dup		

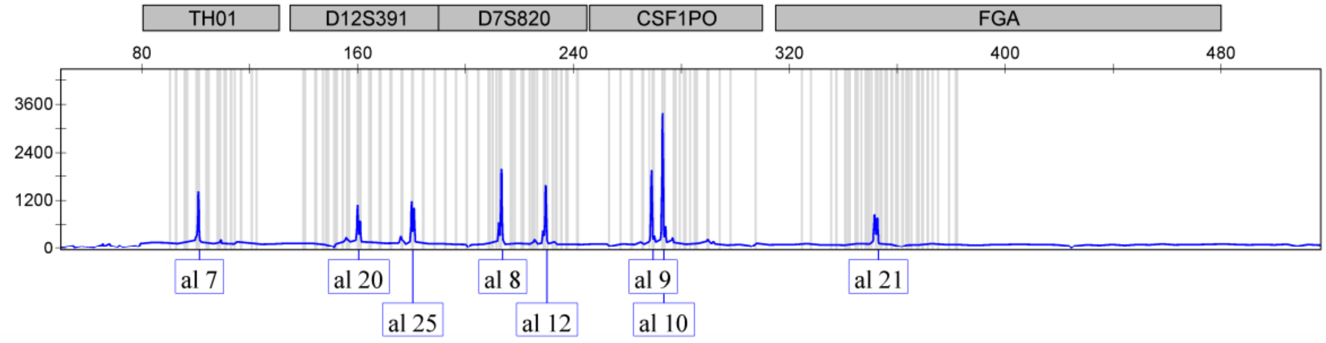

68_D09_XiBaoJianDing(0705+0702)-1-0707.fsa	SMMC-7721	STR Profile 2-dup		

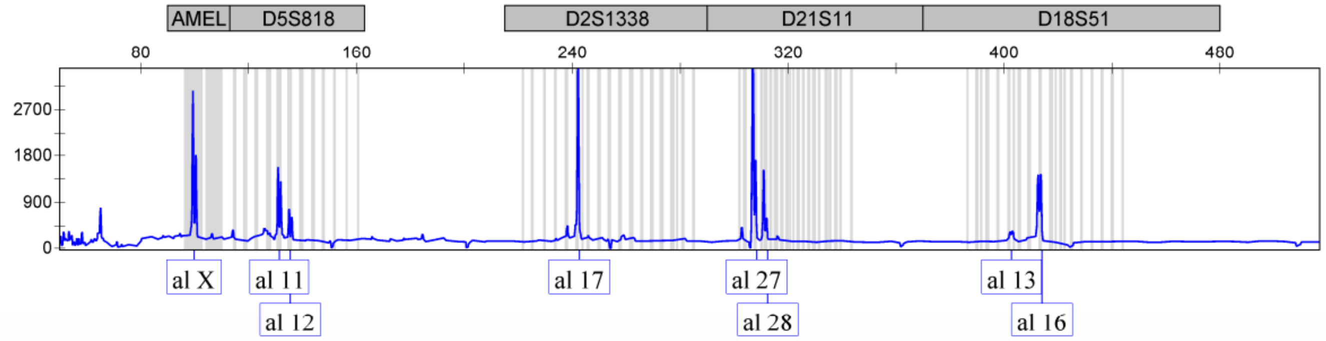

04_D01_XiBaoJianDing(0705+0702)-3-0707.FSA	SMMC-7721	STR Profile 3-dup		

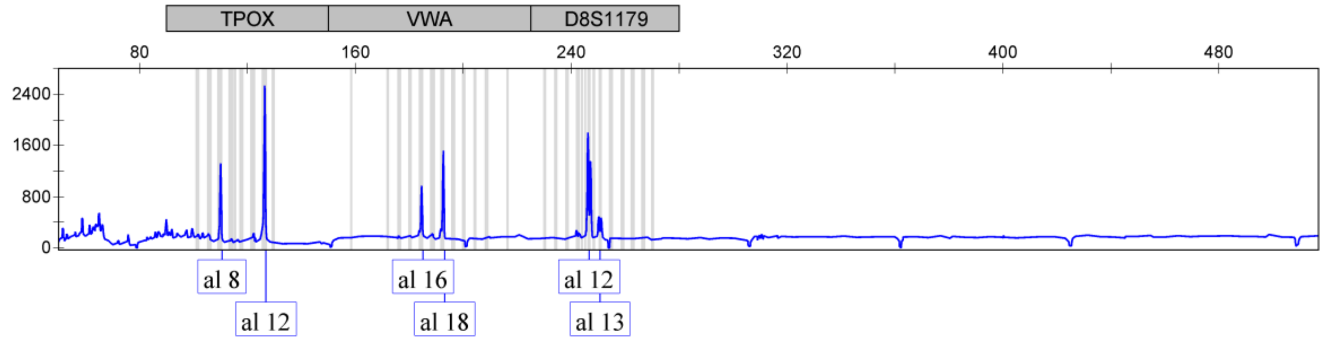

Sample File	Sample Name	Panel	OS	SQ
44_D06_XiBaoJianDing(0705+0702)-3-0707.FSA	SMMC-7721	STR Profile 4-dup		

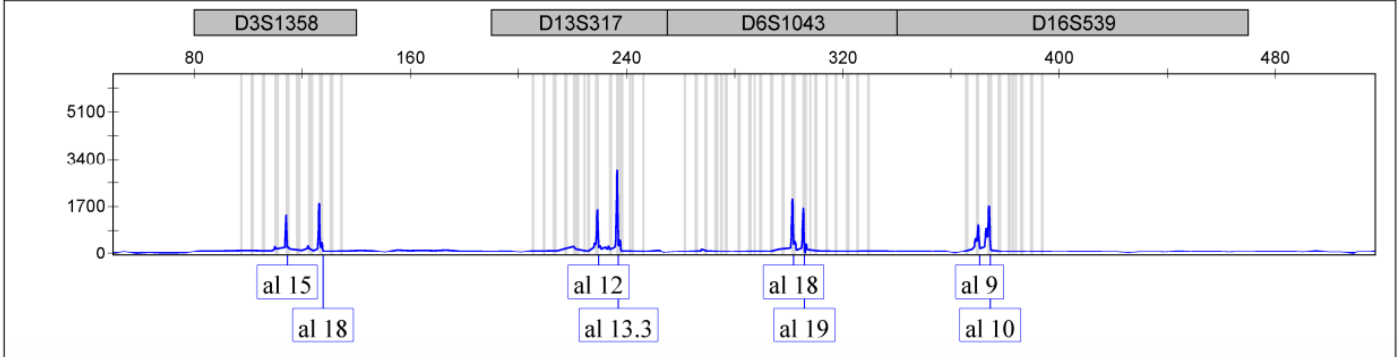

Sample File	Sample Name	Panel	OS	SQ
76_D10_XiBaoJianDing(0705+0702)-3-0707.FSA	SMMC-7721	STR Profile 5-dup		

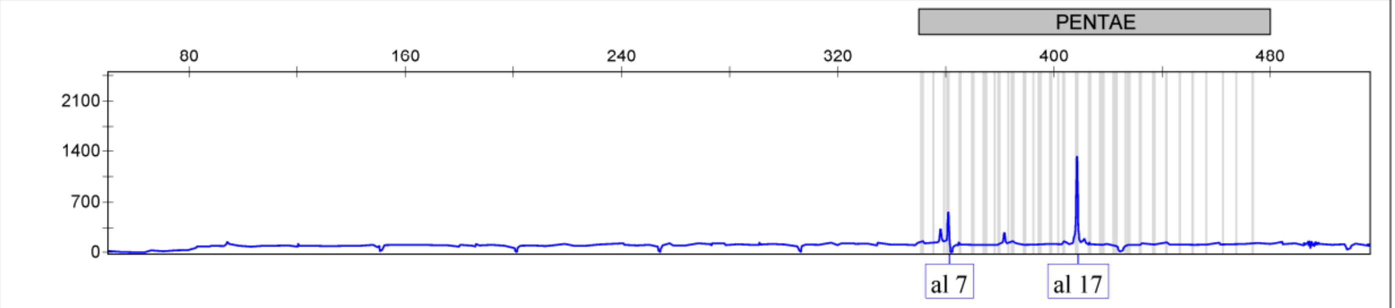

Sample File	Sample Name	Panel	OS	SQ
92_D12_XiBaoJianDing0705+0702FSA	SMMC-7721	STR Profile 6-dup		

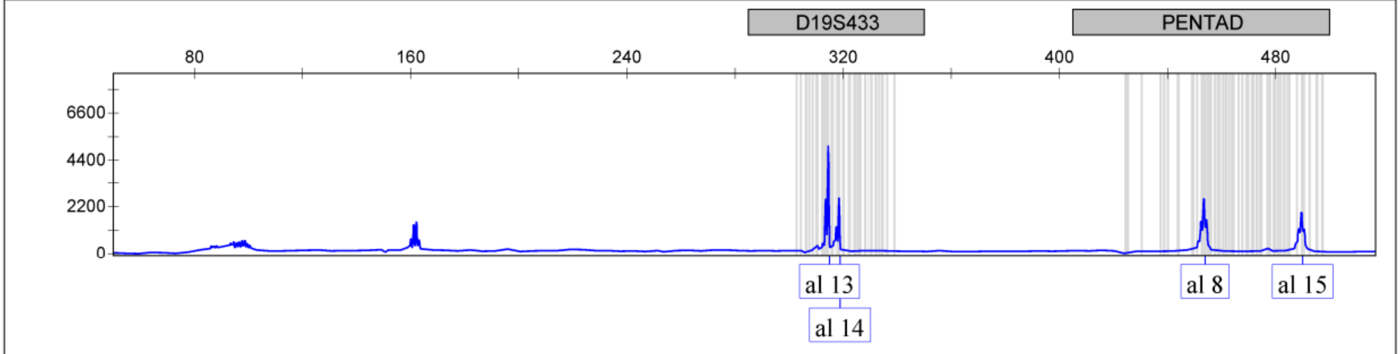

Supplement: Supplementary file 5 [file Data_Sheet_3.pdf]
